# Relationship between Salivary Oxytocin Levels and Generosity in Preschoolers

**DOI:** 10.1038/srep38662

**Published:** 2016-12-08

**Authors:** Takayuki Fujii, Joanna Schug, Kuniyuki Nishina, Taiki Takahashi, Hiroyuki Okada, Haruto Takagishi

**Affiliations:** 1 Brain Science Institute, Tamagawa University, 6-1-1 Tamagawagakuen, Machida, Tokyo, 194-8610, Japan; 2 Department of Psychology, College of William & Mary, Williamsburg, VA, USA; 3 Graduate School of Brain Sciences, Tamagawa University, 6-1-1 Tamagawagakuen, Machida, Tokyo, 194-8610, Japan; 4 Department of Behavioral Science, Hokkaido University, Kita 10, Nishi 7, Sapporo, Hokkaido, 060-0810, Japan

## Abstract

This study examined the association between salivary oxytocin (sOT) levels and generosity in preschoolers. Fifty preschoolers played two dictator games (DG) by deciding how to allocate 10 chocolates between themselves and another child, who was either from the same class as the participant (ingroup member), or an unknown child from another class (outgroup member). sOT levels were assessed in saliva collected from the children immediately prior to the DG tasks. While sOT levels were negatively associated with allocations made to both ingroup and outgroup members by boys, among girl sOT levels were positively related to allocations made to ingroup members, and unrelated to allocations made to outgroup members. These results suggest sex differences in the association between salivary oxytocin and generosity.

Ingroup favouritism is a fundamental psychological mechanism whereby people tend to be more generous and cooperative to ingroup members than to outgroup members^1^,^2^,^3^. Ingroup favouritism is observed in various situations and cultures around the world^4^,^5^,^6^,^7^, and many studies suggest that humans have evolved several tendencies that enable them to quickly identify and cooperate with group members, such as attending to reputation^8^,^9^ and reciprocity^10^,^11^. These ingroup favouring tendencies become adaptive in cases of intergroup conflict (e.g., war)^12^ and may lead to the creation of cultural groups^13^.

Recent studies have started to reveal the biological foundations of ingroup favoritism^14^,^15^,^16^,^17^,^18^. One focus of such research is the role of oxytocin, a peptide hormone produced in the hypothalamus and secreted from the posterior pituitary gland. Oxytocin has been shown to be associated with social cognition, in addition to social stress/anxiety reduction^19^. Recent research suggests that oxytocin plays a pivotal role not only as a hormone but also as a neurotransmitter in the brain, enhancing various aspects of human sociality, such as trusting behaviour^20^,^21^, theory of mind^22^, facial expression recognition^23^, parenting behavior^24^, and social bonding^25^,^26^.

Oxytocin also appears to play a role in increasing ingroup favouritism (for a review, see ref. 27). For instance, De Dreu *et al*.^15^ found that intranasal oxytocin administration enhances individuals’ cooperativeness with other ingroup members when playing economic games. Interestingly, oxytocin did not affect cooperativeness with outgroup members. Another study found that intranasal administration of oxytocin increased the liking of symbols representing national identity^28^, and yet other studies suggest that genetic variants associated with the oxytocin receptor gene (*OXTR*) influence processes related to ingroup favouritism. For instance, one study found that individuals who were homozygous for the G-allele of *OXTR* rs53576 were more likely to show empathetic responses, which were assessed by measuring activity in the anterior cingulate and supplementary motor area in response to racial ingroup members and compared to responses to racial outgroup members^29^. Thus, oxytocin appears to play a role in increasing social bonding and affiliation within groups.

## Sex differences in the effects of oxytocin on social behaviour

A large number of studies have shown that the effects of oxytocin on mammalian social behaviour differ between males and females (for reviews, see refs 30, 31). Oxytocin is implicated in a number of sex-specific process involved in gestation, lactation, and maternal social behaviour in mammalian females, and oxytocin receptors are induced by estradiol, a female sex hormone, which enhances responsiveness to oxytocin^32^,^33^. For instance, studies examining sex differences in the impact of oxytocin on social behaviour in non-human animals have found that intranasal oxytocin administration enhances gazing behaviour toward the owner only in female dogs^34^ and the anxiolytic effect of oxytocin differs between male and female rodents, possibly due to differences in sex steroid hormones^19^.

In humans, similar evidence suggests sex differences in the role of oxytocin on social behaviour. For instance, Rilling and colleagues^35^,^36^ examined sex differences in the effect of intranasal oxytocin administration on cooperative behaviour, and found that oxytocin influenced cooperative behaviour only among female participants. In their study, participants played a repeated prisoner’s dilemma game, and female participants who received the intranasal administration of oxytocin tended to detect others’ betrayal when the opponents were computer-controlled^36^. However, this effect was not observed among male participants^35^. Likewise, Scheele *et al*.^37^ found that oxytocin differentially affected moralistic behaviour in men and women, whereby the administration of oxytocin enhanced self-beneficial choices in a moral dilemma task in males, but inhibited self-beneficial choices in females.

This study sought to examine the relation between oxytocin and social behavior in preschool children. To date, a great deal of research has focused on the emergence of ingroup favoritism in childhood. Recent developmental research has shown that even 6-year-old children can distinguish ingroup members from outgroup members, and tend to be more generous toward ingroup members^38^,^39^,^40^. However, no study has examined the association between ingroup favouritism and oxytocin levels in children. Thus, we sought to examine whether the impact of oxytocin on social behaviour, in this case ingroup favouritism, can be observed in children, and if so, whether there are sex differences in the association of oxytocin to ingroup favouritism.

Investigating the relation between social behavior and oxytocin in children is an important key to understanding the biological development of behaviour related to social norms, as well as developmental disorders. As research has suggested that oxytocin may be implicated in developmental disorders in childhood such as Autism Spectrum Disorders (ASD), examining the development of social behavior in childhood is important to gain a better understanding of how social behavior relates to oxytocin levels. Furthermore, if ASD in children is related to oxytocin levels, we would expect an association between social behavior and oxytocin levels in children.

To assess oxytocin levels, we examined oxytocin concentration in saliva. Recent studies have demonstrated a positive relationship between sociality and salivary oxytocin (sOT) in preschool-aged children^41^,^42^,^43^. Furthermore, because saliva collection is simple and painless, it is better suited to assessing oxytocin levels in children than other methods, such as using plasma. In order to assess generosity shown towards ingroup and outgroup members, we used a simple two-person economic game known as the dictator game (DG), in which children decided how many resources to allocate between themselves and an ingroup and an outgroup member, respectively.

As oxytocin is implicated in social behaviours that are particularly relevant within the context of ingroups familial relationships, we suspected that sOT levels may be positively related to generosity expressed toward ingroup members in particular. Furthermore, as previous studies have found evidence for sex-specific effects of the OT system on social behaviour, we sought to examine whether sex effects would be observed even in preschool children.

## Results

### Behavioural Data

To examine the effect of condition order on allocations made in the DG, we conducted a two-way analysis of variance (ANOVA) with recipient affiliation (ingroup vs. outgroup) as a within-subject factor and condition order as a between-subject factor. Results showed no main effect of the affiliation of the recipient (*F* (1, 48) = 1.35, *p* = 0.2517, *η*_*p*_^2^ = 0.027), condition order (*F* (1, 48) = 0.153, *p* = 0.6971, *η*_*p*_^2^ = 0.003), and an interaction effect (*F* (1, 48) = 3.47, *p* = 0.0688, *η*_*p*_^2^ = 0.067). Thus, we concluded that an effect of condition order on allocations was not observed in this study.

**Figure 1:**
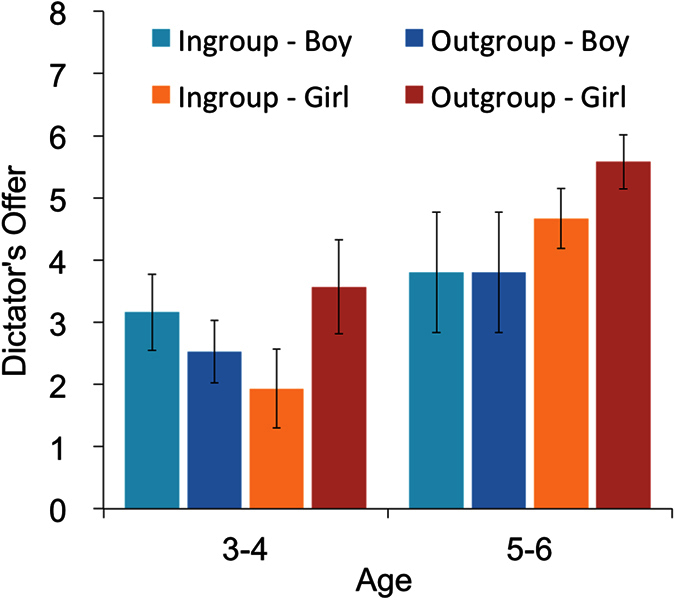
Mean level of the amount of offer in each condition by sex. Error bars represent the standard error of the mean.

The mean levels of the allocation amounts by condition, and sex for older (age 5–6) and younger (age 3–4) children are shown in Fig. 1. We conducted an analysis of covariance (ANCOVA) model on the amounts allocated, with recipient affiliation (ingroup vs. outgroup) as a within-subject factor and sex as a between-subject factor. Because previous studies have shown an effect of age on allocations made by children^44^,^45^,^46^,^47^,^48^, we used age in months as a covariate. Results indicated an interaction effect of recipient affiliation × sex (*F* (1, 47) = 7.30, *p* = 0.0096, *η*_*p*_^2^ = 0.134) and a main effect of age (*F* (1, 47) = 11.34, *p* = 0.0015, *η*_*p*_^2^ = 0.194). However, the main effects of sex (*F* (1, 47) = 0.52, *p* = 0.4736, *η*_*p*_^2^ = 0.011), recipient affiliation (*F* (1, 47) = 0.88, *p* = 0.3523, *η*_*p*_^2^ = 0.018), and the recipient affiliation × age interaction effect (*F* (1, 47) = 0.53, *p* = 0.4708, *η*_*p*_^2^ = 0.011) were not significant. When we added the interaction term of recipient affiliation × sex × age in this model, we did not find significant interaction effect (*F* (1, 46) = 1.63, *p* = 0.208, *η*_*p*_^2^ = 0.034).

### Hormonal Data

First, we examined whether sOT levels were related to sex or age. There was no significant difference in sOT levels between boys (*M* = 3.24 pg/ml, *SD* = 1.52, range = 0.94–6.31, n = 21) and girls (*M* = 3.20 pg/ml, *SD* = 1.65, range = 0.80–6.09, n = 23, *t* (42) = 0.08, *p* = 0.9340, *d* = 0.03), and sOT levels were not correlated with age in months in either boys or girls (boys: *r* = 0.048, *p* = 0.8379; girls: *r* = −0.075, *p* = 0.7342).

Next, we conducted an ANCOVA examining amounts allocated, with recipient affiliation (ingroup vs. outgroup) as a within-subject factor, participant sex as a between-subjects factor, and age in months and sOT levels as covariates. Results showed a significant interaction between sOT level × sex (*F* (1, 39) = 14.12, *p* = 0.0006, *η*_*p*_^2^ = 0.266) in addition to the significant main effects of age (*F* (1, 37) = 10.21, *p* = 0.0028, *η*_*p*_^2^ = 0.207), sex (*F* (1, 39) = 8.59, *p* = 0.0056, *η*_*p*_^2^ = 0.180), and sOT levels (*F* (1, 39) = 4.61, *p* = 0.0381, *η*_*p*_^2^ = 0.106). No significant effects were observed for recipient affiliation (*F* (1, 39) = 0.81, *p* = 0.3728, *η*_*p*_^2^ = 0.020), recipient affiliation × sOT (*F* (1, 37) = 1.22, *p* = 0.2758, *η*_*p*_^2^ = 0.030), recipient affiliation × sex (*F* (1, 39) = 3.91, *p* = 0.0550, *η*_*p*_^2^ = 0.091) recipient affiliation × age (*F* (1, 39) = 0.18, *p* = 0.6778, *η*_*p*_^2^ = 0.004), or recipient affiliation × sex × sOT (*F* (1, 39) = 1.26, *p* = 0.2679, *η*_*p*_^2^ = 0.031) were observed.

**Figure 2:**
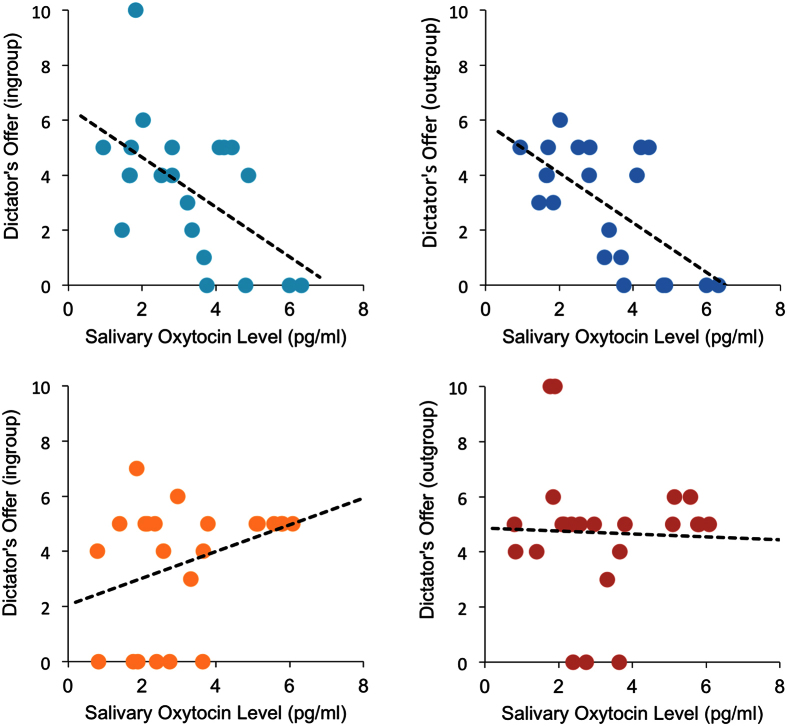
Association between sOT levels and the amount of offer in each condition by sex. In boys, sOT levels were negatively associated with the dictator’s offer in the ingroup (**a**) and outgroup conditions (**b**). In girls, while sOT levels were positively associated with the dictator’s offer in the ingroup (**c**), sOT was not associated with the dictator’s offer in the outgroup conditions (**d**).

As we observed a significant sOT level × sex interaction, we examined the correlations between sOT levels and DG offers made by girls and boys. As the results indicated a main effect of age on allocations, we examined partial correlations controlling for the effect of age. Among boys, sOT levels correlated negatively with allocations made to ingroup (*r* (18) = −0.568, *p* = 0.0090) (Fig. 2a) and outgroup members (*r* (18) = −0.704, *p* = 0.0005) (Fig. 2b). For girls, sOT levels were positively correlated with allocations made to ingroup members (*r* (20) = 0.444, *p* = 0.0387) (Fig. 2c), but not to outgroup members (*r* (20) = −0.016, *p* = 0.9420) (Fig. 2d). Although the recipient affiliation × sOT × sex ANCOVA reported above did not find a significant three-way interaction, a sOT × sex ANCOVA on allocations made to ingroup members with age as a covariate found a significant interaction between sOT and sex (*F* (1, 39) = 13.70, *p* = 0.0007, *η*_*p*_^2^ = 0.260), in addition to significant main effects of sex (*F* (1, 39) = 12.75, *p* = 0.0010, *η*_*p*_^2^ = 0.246) and age (*F* (1, 39) = 7.92, *p* = 0.0076, *η*_*p*_^2^ = 0.169).

Finally, we examined the association between sOT levels and the differences in the allocations made to ingroup and outgroup members, for both boys and girls. The results indicated no significant correlation in the ingroup-outgroup difference scores and sOT levels made by boys (*r* = 0.000, *p* = 0.9989) or girls (*r* = 0.298, *p* = 0.1675).

## Discussion

In this study, we examined the association between sOT level and generosity toward ingroup and outgroup members in preschoolers in the context of a DG. While sOT levels observed in boys were negatively correlated with allocations made to both ingroup and outgroup members, sOT levels in girls significantly correlated with allocations made to the ingroup only. These results suggest that sOT may negatively influence generosity in boys, regardless of whether the target of generosity is an ingroup member or an outgroup member.

While a previous study showed a positive association between oxytocin levels and the extent to which children engaged in social interaction with their parents^42^, to our knowledge this is the first study to show an association between oxytocin level and generosity in children. Although several studies have demonstrated a relationship between oxytocin and ingroup favouritism in adults^14^,^15^,^16^,^17^,^18^, our study did not find such a pattern among preschoolers. Furthermore, this study suggests the existence of sex differences in the influence of oxytocin on generosity in preschoolers. This finding is notable given the number of findings in the literature suggesting that oxytocin may function to influence social behaviour in sex-specific ways^35^,^36^,^37^.

A number of possible neuro-psychological processes may underlie the negative relationship between sOT levels and generosity observed in boys. We suspect that high levels of sOT might be associated with a reduction in social risk taking (i.e., socially defensive tendencies) in boys. Prosocial behaviour, including behaviours such as interpersonal trust, often correspond to social risk taking^49^,^50^,^51^; this may be because prosocial behaviour may be beneficial for oneself, but only in the long run (prosocial/generous behaviour often does not pay off immediately, but is rather returned through reciprocity). In line with this claim, a recent study reported that oxytocin was related to risk aversion in males (but not in females) only under social conditions^52^. Another recent study of preschool children showed that children became more generous when they were monitored by others^53^, supporting the notion that increases in generosity observed across development in childhood may reflect strategic behavior rather than pure increases in prosociality^54^,^55^. That is, preschoolers may expect future reciprocity from those who are aware of their pro-social behavior, and behave in a generous manner when they are observed by others in order to elicit such reciprocal behavior. In this study, because children determined the amounts they wanted to allocate in the DG while they were being watched by the experimenter, children with low sOT levels may have been more willing in engage in risk seeking behavior, and therefore allocated more chocolate to others. Thus, it is possible that the association between sOT levels and amounts allocated by boys in the DG may be reduced in completely anonymous situations.

Another potential interpretation of these results is that oxytocin is associated with increased perception of social competition in boys. This interpretation is consistent with a previous study showing that oxytocin was related to the perception of competitiveness in men, but kinship in women^56^.

In this study, while a significant three-way interaction between SOT, target affiliation, and participant sex was not observed, the direction of the effects for boys and girls were opposite in direction: sOT levels were negatively correlated with allocations to both ingroup and outgroup members amoung boys, and positively correlated with allocations to ingroup members among girls. Future studies should examine whether sOT relates to underlying factors associated with ingroup favoritism that have been shown to vary based on gender, such as social dominance orientation^57^. Furthermore, as it is possible that the current study did not have enough power to reliably show such an effect among girls, future studies should include a larger sample size to investigate sex differences in the effect of oxytocin on generosity in preschool aged children.

Future studies should further examine these and other hypotheses to understand how oxytocin relates to generosity, risk taking, and competition in boys and girls. In addition, because the DG used in this study cannot differentiate between ingroup favoritism and outgroup derogation, future studies should use more diverse paradigms to further investigate the relation between salivary oxytocin and behavior toward ingroup and outgroup members in preschool aged boys and girls.

While the amounts allocated by participants overall in this study are on par with amounts typically offered in DG paradigms by adults participants in identifiable situations^58^, notably, this study did not replicate findings of previous studies reporting ingroup favouritism in children^38^,^39^,^40^, finding no evidence of ingroup favoritism among boys, and evidence of outgroup favoritism in girls. These inconsistent results might stem from the differing experimental situations used across these studies. Although the recipients were randomly chosen from group photos in our study, in a previous study the recipients in the ingroup condition were specific friends of the children^40^. Thus, children in the ingroup condition in this previous study^40^ may have been more strongly motivated to give their own resources to the recipients than were those in our study. Furthermore, paradigms used in previous studies did not incur a cost to participants to provide ingroup members with resources^38^,^39^; this study required children to give up their own resources for the recipients, which may have diminished the desire to provide ingroup members with rewards. Consistent with this hypothesis, Fehr *et al*.^48^ did not find ingroup favouritism in preschoolers using a sharing game similar to the paradigm used in our study, in which sharing was costly to participants. Furthermore, a recent neuroimaging study^59^ showed that the cortical thickness of the dorsolateral prefrontal cortex, an area that plays an important role in executive function, positively relates to both age as well as the inhibition of the desire for economic interests in elementary school-aged children. Thus, it is likely that when children must allocate their own resources in order to provide resources to a recipient, it may be more difficult to observe effects of generosity or ingroup favouritism.

In summary, this study found that sOT levels relate negatively to generosity shown toward both ingroup and outgroup members in boys. Further research is needed to examine sex differences in the association between oxytocin and social behaviour in children, as well as developmental changes in the relation between oxytocin and social behaviour. Future studies should examine the effect of other neuropeptides such as vasopressin, a neuropeptide structurally similar to oxytocin that is thought to moderate a number of social behaviours shown by mammalian males (for a review, see ref. 30). Furthermore, since the ability to measure sOT in saliva is a relatively recent development^60^, it is necessary to confirm whether the findings in our study can be replicated using other oxytocin indicators (e.g., urine sOT levels)^34^,^61^ and genetic variations in oxytocin receptor genes^62^.

## Methods

### Participants

Fifty Japanese preschoolers (26 girls and 24 boys) participated in the study. The mean age in months of participants was 56.9 (*SD* = 11.6, range: 38–81). The experimenter explained the outline of the research to all children and their guardians. Informed consent was obtained from guardians of all participants, and participants provided their assent to take part in the study. The ethical committee at Tamagawa University approved this study and the methods in this study were carried out in accordance with the approved guidelines.

### Dictator Game

**Figure 3:**
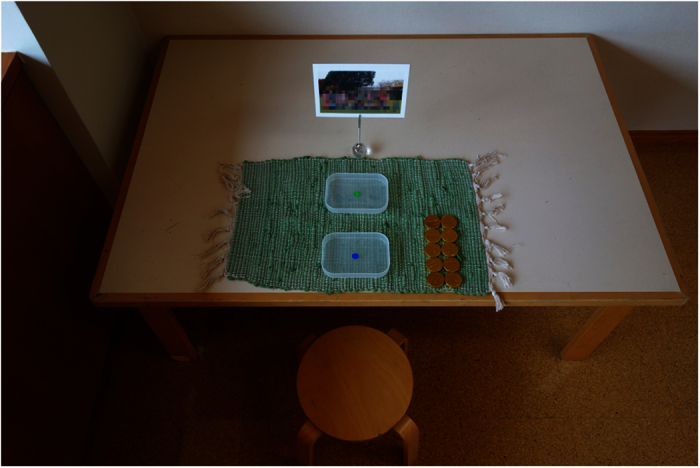
A photo of the experiment environment. Children sat at a desk and decided how to allocate ten chocolates. A male experimenter sat next to the child and explained the rules of the game to the children.

Children played the DG in a vacant room with a male experimenter present. The experimenter sat next to the child and explained the DG to him/her. After the description, the experimenter confirmed the child’s understanding of the game. Following this confirmation, the child decided how to allocate 10 coin chocolates between him/herself and another child (the recipient). The recipient’s affiliation was manipulated such that participants played either with an ingroup member (a child from the same class) or an outgroup member (an unknown child from a different class). A group photo was placed on the table and the children were told that the recipient would be randomly chosen from the members in the group photo after the experiment (Fig. 3). In the ingroup condition, a photo of the participant’s classmates was placed on the table. However, in the outgroup condition, a photo of preschoolers who attended a different kindergarten from the participants was placed on the table. Both pictures included both boys and girls. These two conditions were assigned as a within-subjects factor, and the order of these conditions was randomized and performed subsequently. The task was completed once children had decided how many chocolates to allocate to another child in both conditions.

### Collection and Assessment of Salivary Oxytocin

In order to assess salivary oxytocin levels, we collected saliva from participants with the Saliva Collection Aid (Salimetrics, Inc., State College, PA) using the passive drool method. Children were asked to produce at least 1 ml of saliva, which was collected in a cryogenic vial (2 ml). A total of six children were unable to produce more than 1 ml of saliva, resulting in a total of 44 saliva samples. Saliva was collected before the DG between 9:00 am and 11:00 am. Cryovials containing the saliva were immediately frozen in a storage box and stored at −80 °C until assay, which was conducted at a professional analysis agency (MACROPHI Inc., Japan, http://www.macrophi.co.jp/english/index.htm). The process of preparing each sample was as follows: First, the sample (1 ml) underwent column-based purification. Second, the column-purified liquid was frozen at −80 °C and freeze-dried. Third, the sample was lyophilized by adding an Assay Buffer (concentration ratio was four times). Finally, the sample was centrifuged for 5 min (800 × g) and the supernatant was subjected to ELISA assay. The assay was conducted using a commercially available Oxytocin ELISA kit (Enzo Life Sciences, Inc., Farmingdale, NY). Each sample was prepared in duplicate, and concentrations were calculated using the SpectraMax^®^ (Molecular Device, Sunnyvale, LLC, Sunnyvale, CA) microplate reader according to relevant standard curves.

## Additional Information

**How to cite this article:** Fujii, T. *et al*. Relationship between Salivary Oxytocin Levels and Generosity in Preschoolers. *Sci. Rep.* **6**, 38662; doi: 10.1038/srep38662 (2016).

**Publisher's note:** Springer Nature remains neutral with regard to jurisdictional claims in published maps and institutional affiliations.
